# Newspaper depictions of mental and physical health

**DOI:** 10.1192/pb.bp.116.054775

**Published:** 2017-12

**Authors:** Marian Chen, Stephen Lawrie

**Affiliations:** 1 Medical School, University of Edinburgh; 2 Division of Psychiatry, University of Edinburgh

## Abstract

**Aims and method** Media portrayals of mental illness have long been recognised as being misleading and stigmatising. Following the campaigns of several advocacy groups to address this issue, we aimed to evaluate the impact on mental health reporting over time. We repeated a survey we did 15 years ago using the same methods. Nine UK daily newspapers were surveyed over a 4-week period and coded with a schema to analyse the reporting of mental health compared with physical health.

**Results** In total, 963 articles – 200 on mental health and 763 on physical health – were identified. Over half of the articles on mental health were negative in tone: 18.5% indicated an association with violence compared with 0.3% of articles on physical health. However, there were more quotes from patients with mental disorders than physical disorders (22.5% *v.* 19.7%) and an equal mention of treatment and rehabilitation.

**Clinical implications** Mental health in print media remains tainted by themes of violence, however some improvement in reporting in recent years is evident, in particular by providing a voice for people with mental illness.

Newspapers and other forms of mass media are influential in transmitting key information and ideas, including social commentary.^1,2^ Consequently, their role in reporting on mental illness is seen as one of the main conduits through which the public, especially those without personal exposure to mental illness, learn and come into contact with issues surrounding mental health.^3,4^

For many years it has been recognised that media reporting of mental illness is heavily biased in representing a misleading link between mental illness and violence, with preferential reporting for sensationalist stories depicting individuals with mental disorders as being aggressive, dangerous and unpredictable.^1,4–7^ Such widespread negative portrayals have reinforced the continued stigmatisation and lack of acceptance among those with psychiatric diagnoses, possibly increasing social distance and contributing to morbidity and distress. Of particular concern is the potential ‘internalisation’ of prejudices portrayed by the media, such that people with mental illnesses also begin to take on the view that they are dangerous and unstable.^8^ Furthermore, there is a corresponding lack of voice for people of mental illness,^6,8^ with few personal accounts of their burden of disease and similarly little representation from qualified healthcare professionals.^6,9^ All of this contributes to creating a stereotyped, isolated group without social status and acceptance.^10^

In a similar vein, there is a disappointing lack of coverage of stories on recovery and rehabilitation following mental illness.^5,6^ This potentially leads both the public and policy makers to erroneously believe that recovery is rare. We highlighted this in a newspaper survey 15 years ago in which we found that the reporting of mental illness and people with mental illness tended to be negative, whereas that of physical illness was much more likely to be positive in tone.^11^ This may even influence health resource allocations to conditions that have a higher perceived chance of cure.^4^

As far back as the 1960s, Nunally noted that mental health representations in media were few and distorted, limited to ‘neurotic behaviour’ in evening dramas where ‘the afflicted person often enters the scene staring glassy-eyed, with his mouth widely agape, mumbling incoherent phrases or laughing uncontrollably’.^12^ Steadman & Cocozza^13^ also showed that media depictions are often skewed towards extremes. Their findings on public perception of the ‘criminally insane’ (mentally disordered offenders) are salient, with 61% of the public surveyed responding that they feared former mentally disordered offenders ‘a lot’.^13^ However, as the authors noted, the mentally disordered offenders named by the public following intensive coverage tend not to have any legal claim to be ‘criminally insane’, and are merely assumed to be as such by the media.

Although those with mental illness are reportedly 2–6 times more likely to commit violence than matched controls,^5,14^ the National Confidential Inquiry into Suicide and Homicide by People with Mental Illness^15^ found that only 5% of the homicides carried out in the general population between 2001 and 2011 were by those with an abnormal mental state (*n* = 52). However, an additional 14% had been in contact with mental health services in the preceding 12 months. Therefore, the vast majority of perpetrators of homicide do not have a mental illness at the time of the offence (*n* = 951). Contrast this with the statistics for suicide in the same report: 30% of all completed suicides in the 10-year period analysed were by patients already known to mental health services (*n* = 2678). The figures identified are 14 times higher than for homicide. From this, it is reasonable to infer that an individual with a mental health disorder is far more likely to self-harm than harm others. This important point is also often missing from media reports seeking to capitalise on the sensationalist angle and, by doing so, dehumanise the patient to the extent that they are defined by their illness. Similarly, it is clear that people with mental illness are much more likely to be ‘victims’ of crime than perpetrators.^16,17^ This discrepancy in public perception between perpetrator and victim among those with mental illness has also been highlighted in other studies,^18,19^ acknowledging the need to shift attention away from the perceived threat to the public, to providing support and protection for the truly vulnerable. In this regard, the media has been counterproductive in reinforcing misleading stereotypes about public safety while simultaneously underreporting key statistics on mental health.

Although viral campaigns such as Rethink Mental Illness’ ‘Find Mike’^20^ have been highly successful in bringing mental illness to some public attention, it is difficult to assess their impact. Based on our previous survey,^11^ we hypothesised that: mental health reporting would still be consistently more negative in tone compared with reporting of physical health issues, and patients would be consistently underrepresented in media depictions compared with physical health reports. Additionally, regarding the link with violence and criminality, we further hypothesised that the association between mental illness and violence would continue to pervade mental health reporting.

## Method

The method followed that of Lawrie.^11^ Nine weekday UK daily newspapers (Scottish editions) were surveyed over a period of 4 weeks between 4 March and 4 April 2014. The papers chosen represent a selection of popular broadsheet (*The Times, The Telegraph, The Independent, The Guardian, The Scotsman, The Herald*) and tabloid (*Daily Mail, The Sun, Daily Record*) publications in the UK, with a reported range in circulation of between 64 279 (*The Independent*) and 2 048 977 (*The Sun*) in February 2014^21^ Only national newspapers were included in this analysis, as they have the widest distribution.

Print copies of the newspapers were obtained from the Reference Library of Edinburgh on the day of publication and manually screened for headlines relating to mental health or psychiatry and general health issues. Two tabloids, *The Sun* and *Daily Record*, were purchased each day, as they were not subscribed to by library services, yet have the highest circulation figures in the UK.

Articles relating to mental health and physical health were identified initially through headline screening. The main body of text was then read and coded as positive, negative or neutral, if the article was mainly stating facts or had mixed elements. As such, it is possible to have a disparity between tone of headline and article. Each article was then analysed and coded to a schema with eight variables, which noted key themes (Table 1).

**Table 1:** Coding schema for newspaper articles

Variable	Description
Quote from patient, relative or charity	Direct or paraphrased quote from a patient with the illness in question, relative or advocacy organisation

Quote from professional	Quote from a healthcare professional in charge of the patient's care or spokesperson for government organisations, etc.

Association with violence	Any article where a person portrayed as having a mental illness or under the influence of drugs/alcohol engages in criminal or dangerous activity where others are put at risk or harmed. Suicide or self-harm is not included in this category

Mention of treatment/ rehabilitation	Either specific (e.g. Prozac), general (anti-depressants, cognitive–behavioural therapy), alternative (mindfulness, yoga) or relating to the Mental Health Act 1983 where treatment is noted under a compulsory treatment order

Substance misuse	Articles relating to addiction, overdose or psychiatric effects of substance misuse were classified as relating to mental health. Physical effects of drug usage, including side-effects, were classified under physical health

System failure/ negligence	Any article where substandard care is delivered by a single person or on an organisational level, resulting in adverse outcomes. Shortcomings range from IT failures and changes in social policy to deliberate mistreatment

Innovations in research	Articles where a new diagnostic method, treatment or better understanding of a named disease is reached

Medical advocacy/raising awareness	Where the primary purpose of the article is to transmit information about a particular disease profile or set of symptoms or charity efforts. For example Autism Awareness Week, United Nations World Down Syndrome Day, ‘Check ’Em Tuesday’ – a campaign run by *The Sun* to increase uptake of self-screening for breast cancer

### Inclusion and exclusion criteria

The sports and business sections were surveyed, however property and automobile inserts were excluded due to likely lack of relevance. Primary newspaper articles, editorials and opinion pieces were included in the data collection. Advertisements, letters to the editor, film and entertainment reviews and obituaries were omitted. Articles with only a passing or chance reference to mental or physical health were also omitted.

All articles concerning mental disorders recognised by the DSM-5,^22^ psychiatry or psychiatrists were included, as well as generic, undefined mental conditions with the exception of paedophilia and the paraphilias, as their classification as a mental illness in DSM-5 is controversial. For physical health, all articles relating to other general medical issues were collected. Articles concerning the National Health Service (NHS) or other organisational bodies were included only if there was a demonstrable mention of impact on patient health and safety.

## Results

In total, 963 health-related articles were collected during the 4-week period. Of these, 763 (79.2%) concerned physical health, whereas 200 (20.8%) were about mental health. Although there was a slight preponderance towards neutral pieces (38.0%) in the physical health category, compared with positive (28.6%) and negative (32.9%) reporting, the majority of articles concerning mental health were negative in tone (50.5%), with only a small number of positive (22.0%) or neutral articles (27.5%) (Box 1). This striking disparity between mental and physical health reporting underscores a key finding that mental health reporting remains consistently more negative than physical health, which is in line with the results from Lawrie's^11^ study over a decade previously (ratio of positive to negative articles for physical health: 218/251; and for mental health: 44/101; odds ratio (OR) 1.99, 95% confidence interval (CI) 1.34–2.97). In addition, we found a prominent and stigmatising association between mental illness and violence as 18.5% of mental health articles featured violence as a major theme, compared with 0.3% of physical health articles (OR = 86.37, 95% CI 20.60–362.1) (Table 2).

**Table 2:** Comparison of variables between physical and mental health reporting

Variable	Physical health, *n* (%)	Mental health, *n* (%)
Total number of articles	763	200

Positive	218 (28.6)	44 (22.0)

Negative	251 (32.9)	101 (50.5)

Neutral	290 (38.0)	55 (27.5)

Quote from patient/charity	150 (19.7)	45 (22.5)

Quote from professional	285 (37.4)	58 (29.0)

Association with violence	2 (0.3)	37 (18.5)

Mention of treatment/rehabilitation	217 (28.4)	48 (24.0)

Substance misuse	31 (4.1)	22 (11.0)

Medical advocacy/raising awareness	131 (17.2)	26 (13.0)

System failure/negligence	118 (15.5)	25 (12.5)

Innovations in research	184 (24.1)	22 (11.0)

Although previous studies have shown that patients with mental illness are underrepresented in newspaper articles,^6,8^ we found this not to be the case, with a higher proportion of quotes from patients with mental illness, their relatives or mental health charities (22.5%) compared with physical health (19.7%) (ratio of quotes in physical health: 150/613; mental health: 45/155; OR=0.84, 95% CI 0.58–1.23). There appears to be comparatively fewer quotes from professionals in relation to mental health however, highlighting the need of the psychiatric profession to make a conscious effort to engage with the media, and vice versa.^23^ Interestingly, whereas previous studies have highlighted a lack of reporting on treatment and rehabilitation for mental health,^4–6^ here we found the rates to be comparable to that of physical health articles (24.0% *v.* 28.4% respectively) and previous reports.^6^

**Box 1** Examples of positive, negative and neutral headlines for mental and physical health

**Physical health**

**Positive**

‘US Aids baby may be cured’

‘Cholesterol drugs may slow MS, study shows’

**Negative**

‘Three-year old died after hospital delay’

‘ “Sickness culture” claim as 1 in 3 has long-term illness’

**Neutral**

‘Statins have no side-effects, study finds’

‘Smoking link to breast cancer in older women’

**Mental health**

**Positive**

‘Depression and self-harming are taboo … that's why I'm determined to speak out’

‘Ketamine trial provides fresh hope for long-term depressed’

**Negative**

‘Mentally ill man in bus knife attack’

‘School accused of excluding girl with anorexia to avoid copycats’

**Neutral**

‘Give dementia patients trial drugs en masse’

‘Depression must be seen as a societal disorder’

The disorders most commonly associated with violence were personality disorders as well as undefined ‘mental illnesses’ and post-traumatic stress disorder, which make up 13.5% of all articles pertaining to mental health. Surprisingly, sensationalist stories about schizophrenia were minimal and comparable to reporting on other mental disorders, whereas dementia and eating disorders were least likely to be reported as associated with violence (Table 3).

**Table 3:** A breakdown of newspaper articles according to diagnosis and association with violence

Diagnosis	Articles *n* (%)	Association with violence, *n* (%)
Suicide	31 (15.5)	0

Mood disorder	26 (13)	2 (1.0)

Dementia	32 (16)	0

Eating disorder	12 (6)	0

Developmental disorder	17 (8.5)	3 (1.5)

Substance misuse	13 (6.5)	2 (1.0)

Personality disorder	11 (5.5)	14 (7.0)

Schizophrenia/psychosis	10 (5)	3 (1.5)

Other^a^	49 (24.5)	13 (6.5)

a. Includes multiple disorders, undefined and post-traumatic stress disorder.

Based on the previous data that show people with mental illnesses are more likely to be victims of crime rather than perpetrators, we were interested to see whether this correlated with media reports. We found that people with mental illness are twice as likely to be portrayed as a perpetrator (34/200) compared with a victim (17/200), in direct contrast to the available data.^16,18,19,24^

Given that 18.5% of articles on mental health concern violent acts and are seen as a threat to the public, we asked what made up the rest of the negative reporting. On further analysis, it appears that a significant proportion of the negative articles pertain to the stigmatisation and abuse of people with mental health within society, as well as lack of funding and adequate care provision. Examples include headlines such as: ‘The great mental health betrayal’ (*The Independent*, 13 March 2014), an article about the unlawful detaining of people with mental health illnesses in care homes and hospitals, and ‘Mental health and race – the blight of dual discrimination’ (*The Guardian*, 26 March 2014), which highlights the additional burden of being an ethnic minority with mental illness. The remainder of the negative articles are reports on suicide (15.5%).

Overall, there was no significant difference between mental health reporting in broadsheets compared with tabloids.

## Discussion

We found that mental health reporting makes up a fifth of all articles concerning health in a selection of UK print media over the course of a month. This is lower than found in a similar study by Pieters *et al*^25^ in Belgium but comparable to the figures obtained by Lawrie in the UK^11^ over a decade ago, reflecting little change in the frequency of reporting on mental health. Half of all articles on mental health are negative in tone, which is a much greater proportion than for physical health, but nonetheless represents a small reduction compared with Lawrie's study. On the other hand, the association with violence remains prominent, with 18.5% of articles carrying a theme of danger or criminality.

In the intervening 15 years, several campaigns by mental health charities both in the UK and abroad have sought to lessen the stigma attached to mental illness and encourage open dialogue about mental health. Media coverage is important to the success of such campaigns in influencing public opinion and increasing exposure. In light of this, we asked whether there is any evidence that media reporting on mental illness is improving (see below). Although previous studies have commented on the lack of voice for people with mental illness, we have found this not to be the case with a higher proportion of quotes from patients with mental illnesses compared with physical health. Although there are fewer representations of healthcare professionals in psychiatry, this may reflect reluctance among the profession as a whole to engage with the media, as psychiatrists too are affected by stigma.^26,27^

### Mental illness and violence

The link between mental illness and violence is unfortunately still pervasively reported – being part of about a fifth of all articles on mental health. The figure obtained in this study (18.5%) is lower than previous reports of association with violence.^6^ However, other studies have utilised more restricted search criteria, only using specific keywords such as ‘schizo’, ‘mental’, ‘deranged’ and ‘psycho’, which does not encompass the whole spectrum of mental illnesses.^5,6,28,29^ As most studies also used a computerised database programme (such as the LexisNexis) to generate articles, limiting the search criteria to only the above keywords will bias the results to find articles on schizophrenia or those that include derogatory terms such as ‘psycho’ or ‘deranged’, which are inherently stigmatic and more likely to be associated with violence.^29^ In contrast, mental illnesses not usually considered violent, such as eating and mood disorders, would be excluded from the analysis.

In our study we found that schizophrenia and psychotic disorders make up a minority (5%) of reporting on mental illness and only 1.5% of these articles featured violence. Although there is little contention that mental disorders are disproportionately associated with violence in the media, one possible explanation for the strength of the association between the two is that it is an artefact created by selective searching among newspaper databases. We suggest the effect would be diluted if all articles on psychiatry, including conditions such as mood and anxiety disorders, were incorporated in the analysis.^30,31^

In this study, we also found that people with mental disorders are twice as likely to be portrayed as a perpetrator of crime compared with a victim. This is another illustration of how the media can be misleading on key facts. Education for journalists and guidance on the reporting of mental health issues is central to bringing about a change in reporting trends. However, this may be easier said than done. One study attempted to correct journalists' erroneous views about mental illness with a short presentation on schizophrenia prior to a press conference regarding a high-profile assault carried out by a man with a serious mental illness. Although they found that journalists had a good recall for the details several weeks later, the presentation did not ultimately affect the writing of the article.^32^ This suggests that collaborations with the media will have a greater chance of success and change in reporting if they are mutually beneficial and desired. One example of this is UK mental health charity, Mind, which has set up a series of media awards for responsible and ethical reporting of mental health issues (www.mind.org.uk/news-campaigns/mind-media-awards), as well as reporting guidelines for different conditions.^33,34^

### Strengths and limitations

The main strength of the study is that all papers were hand-searched according to the criteria set out in the Method. This abolishes the need to search based on keywords, potentially increasing the yield of articles. Similarly, the scope and definition of ‘mental illness’ is wider than in previous studies, allowing for a greater overview of the subject in media. In the current study, the use of one investigator to locate and rate the articles, is both a potential advantage – in reducing interrater variability – but also a disadvantage in that no interrater reliability could be established (although we did discuss and resolve any uncertainties between the authors). The main limitation is that the coding schema used is a relatively blunt tool and cannot convey the complexity or depth of a newspaper story. Second, the categorisation of articles into mental and physical health does not allow for overlap in coverage, although that only occurred in a small minority of articles. Furthermore, the short time frame of the study means it can only provide a snapshot of mental health reporting in the UK. Last, there were two mental health stories that garnered a considerable amount of media attention in this time period and subsequently have the ability to bias the data. One was the suicide of a famous fashion designer and another, the death of a woman who had murdered her three children and was subsequently labelled as having a personality disorder. Both stories were extensively reported on over multiple days. However, they were included in the analysis as this study was designed to encompass all the news on mental health over 4 consecutive weeks, rather than the sampling of random time points, and different reports had different foci as new information about the cases came to light.

### Conclusions

Despite several prominent government-led and charity campaigns, mental illness and individuals with mental illness continue to experience stigmatisation and stereotyping. Although it is encouraging that people with mental disorders are being increasingly represented in print media, and there appears to be an overall increase in non-stigmatising articles,^35^ much remains to be done to address the inaccurate and stigmatising association with violence, and raise the profile of mental illness as a whole.

The particular reasons for the ongoing negative headlines about mental illness, and what could or should be done about them, are beyond the scope of this study. Our results do however indicate that that there is a clear need for more reporting about mental health in general, and for more accurate reporting about violence and for more accounts of innovative research developments in particular. We therefore suggest that there is much to be gained from further ongoing engagement between patients and psychiatrists and the media, with an emphasis on realistic depictions of the lives patients lead and of recent research advances.
